# Understanding the Biology of Antigen Cross-Presentation for the Design of Vaccines Against Cancer

**DOI:** 10.3389/fimmu.2014.00149

**Published:** 2014-04-08

**Authors:** Cynthia M. Fehres, Wendy W. J. Unger, Juan J. Garcia-Vallejo, Yvette van Kooyk

**Affiliations:** ^1^Department of Molecular Cell Biology and Immunology, VU University Medical Center, Amsterdam, Netherlands

**Keywords:** cross-presentation, dendritic cells, antigen processing and presentation, anti-cancer vaccine, CD8^+^ T cells

## Abstract

Antigen cross-presentation, the process in which exogenous antigens are presented on MHC class I molecules, is crucial for the generation of effector CD8^+^ T cell responses. Although multiple cell types are being described to be able to cross-present antigens, *in vivo* this task is mainly carried out by certain subsets of dendritic cells (DCs). Aspects such as the internalization route, the pathway of endocytic trafficking, and the simultaneous activation through pattern-recognition receptors have a determining influence in how antigens are handled for cross-presentation by DCs. In this review, we will summarize new insights in factors that affect antigen cross-presentation of human DC subsets, and we will discuss the possibilities to exploit antigen cross-presentation for immunotherapy against cancer.

## Introduction

For the induction of antigen-specific CD8^+^ T cells, antigen needs to be presented in MHC class I molecules in order to be recognized by the TCR/CD3 complex on CD8^+^ T cells. Peptides derived from endogenous proteins degraded in the cytosol, that are transported into the endoplasmic reticulum (ER), are loaded on MHC class I molecules, which will be transported to the plasma membrane as a stable peptide–MHC class I complex (1). The presentation of endogenous-derived peptides allows the immune system to detect cells that present altered self peptides or foreign peptides and is therefore an important defense mechanism against cancer or viruses (2). Although peptide–MHC class I complexes can be directly recognized by naïve CD8^+^ T cells, these cells require adequate co-stimulation from antigen-presenting cells (APCs) in order to become potent effector CD8^+^ T cells with cytotoxic potential. Besides, APCs can also encounter exogenous antigens, namely of microbial or tumor origin, which they internalize for processing and presentation in MHC class I molecules, a phenomenon known as antigen cross-presentation.

Although multiple APCs are able to cross-present antigens, dendritic cells (DCs) are the most efficient cells *in vivo* (3–5). The potential of DCs to cross-present antigen has initiated many research questions aimed at finding strategies to enhance cross-presentation of DCs in order to improve tumor- and viral-specific CD8^+^ T cell responses for the treatment of cancer or infectious diseases. Several questions remain unanswered, such as the molecular basis for the differences in cross-presentation efficiency observed amongst different DC subsets, in steady-state or under inflammatory conditions. In addition, recent studies also suggest that the capacity to cross-present can be influenced by the type of antigen and the presence and timing of inflammatory signals (6). This would imply that antigen cross-presentation is not a functional specialization of certain DC subsets, but a process that can occur in many APCs under specific conditions. In this review, we will discuss the factors that have been described to influence cross-presentation of various human DC subsets, and their implication in the design of immunotherapies against cancer.

## Cell Biology of Antigen Cross-Presentation

A defining aspect of the adaptive immune system is its capacity to elicit antigen-specific cellular immune responses by the instruction of antigen-specific CD4^+^ and CD8^+^ T cells. This property is entirely based on the presentation of antigen in MHC molecules (the peptide–MHC complex) and its recognition by the T cell receptor. The loading of extracellular antigen in MHC-II, recognized by CD4^+^ T cells, occurs in a different intracellular compartment than the loading of antigen in MHC-I, recognized by CD8^+^ T cells. In the case of MHC-II, after its synthesis in the ER, complexes are formed with CD74 (also known as the invariant chain) to allow proper folding, trafficking, and protection of the peptide-binding groove. CD74 helps guiding the CD74–MHC-II complex move on to the endolysosomal pathway, where late endosomal proteases such as cathepsin S and L degrade CD74 and leave MHC-II complexed to the peptide-binding groove part of CD74 (the CLIP peptide), which is later exchanged for an antigenic fragment with the help of the chaperone HLA-DM (7). Although the process leading to antigen presentation on MHC-I also involves six basic steps (8); namely, acquisition of antigens (1); tagging of the antigenic peptide for destruction (2), proteolysis (3), transport of peptides to the ER (4), loading of peptides to MHC-I molecules (5), and the display of peptide–MHC-I complexes on the cell surface (6); the variety of intracellular compartments and pathways involved in MHC-I antigen presentation is considerably more complex than that of MHC-II.

**Figure 1:**
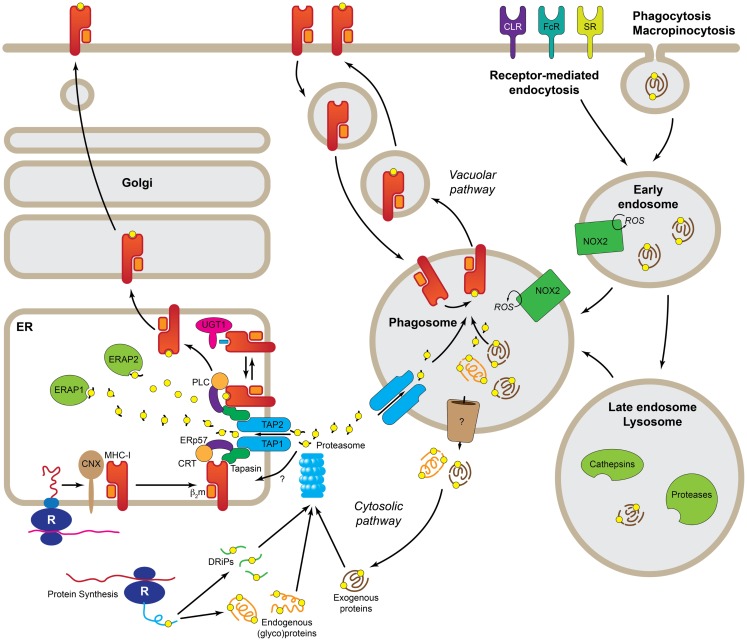
**Molecular pathways leading to cross-presentation in DCs**. DCs take up Ag by three general mechanisms, receptor-mediated endocytosis, phagocytosis, or macropinocytosis. Once the Ag reaches the endolysosomal pathway, depending of the specific routing, it may be degraded by the concourse of the mild pH and different types of cathepsins and other proteases. At this point, properly degraded Ag can be directly loaded into recycling MHC-I in the phagosome (*Vacuolar* pathway). Ag that still needs further processing must be transported to the cytosol (*Cytosolic* pathway) where it is degraded, together with endogenous proteins and DRiPs, by the proteasome. The peptides generated by the proteasome are transported by TAP or a yet uncharacterized transporter into the ER where they are loaded into MHC-I with the help of the peptide-loading complex. Further trimming in the ER prior to loading, it is possible by the presence of ER-localized endopeptidases (ERAP1 and 2). R, ribosome; CNX, calnexin; CRT, calreticulin; b2m, b2microglobulin; UGT1, UDP-glucose:glycoprotein glucosyltransferase 1; ERAP1/2, ER-aminopeptidases 1/2; PLC, peptide-loading complex; ERp57, protein disulfide isomerase 3; TAP1/2, transporter associated with antigen-presenting 1/2; DRiPs, defective ribosomal products; ROS, reactive oxygen species; NOX2, NADPH oxidase 2; CLR, C-type lectins; FcR, Fc receptors; SR, scavenger receptors.

The acquisition of antigenic peptides for MHC-I presentation is a highly heterogeneous process and multiple pathways have been described so far. There are two main sources of antigens for MHC-I presentation, intracellular and extracellular (Figure 1). Antigenic peptides derived from cytosolic proteins, e.g., viral proteins, are the prime source of peptides for MHC-I (9), but other proteins carrying signal sequences targeting to the secretory pathway can also be presented on MHC-I, either from defective ribosomal products (or DriPs) (10) or from mature proteins (11). These mechanisms are at play on all cells expressing MHC-I. However, what makes DCs and, to a lesser extent also macrophages and B cells, best at cross-presentation is their capacity to use extracellular antigens as source of peptides for MHC-I presentation. The uptake of extracellular antigens by APCs is achieved by three main transport pathways, namely receptor-mediated endocytosis, phagocytosis, and macropinocytosis; although there are differences in the efficiency of each of these pathways amongst DCs, B cells, and macrophages. Thus, macrophages seem to be best at phagocytosis, whereas DCs prefer receptor-mediated endocytosis. Amongst the many classes of receptors that mediate endocytosis of antigens are the B cell receptor (specific for B cells), Fc receptors, heat-shock protein receptors, scavenger receptors, and the C-type lectin receptors (CLRs). In general, these receptors mediate internalization of antigens to endosomes, however, the nature of the endosomes and their fate seems to vary for the different receptor types involved and, consequently, also their efficiency in inducing cross-presentation. Furthermore, many of the receptors involved in antigen uptake for cross-presentation are also able to mediate signaling and, in several cases, it has been demonstrated that signaling is necessary for cross-presentation. This was elegantly demonstrated in experiments where bacteria were opsonized with either antibodies or complement. Although both opsonization modalities lead to efficient phagocytosis, only the Fc receptor-mediated resulted in effective CD8^+^ T cell responses (12). Signaling through other receptors, such as the C-type lectins, Dectin-1 (13) or DNGR-1 (also known as Clec9A) (14) also enhances cross-presentation.

Both endogenous and to a minor extent exogenous antigen can thus be loaded on MHC class I. A factor that conditions the access of peptides to MHC-I is the biosynthetic pathway of the MHC-I molecule. The MHC-I complex consists of a heavy chain, a transmembrane glycoprotein with a short cytoplasmic domain that, upon translation in the ER, assembles with β_2_-microglobulin into a heterodimer. This process is integrated with the incorporation of the peptide into the peptide-binding groove of the heavy chain, and requires the participation of the peptide-loading complex, which consists of multiple components, including the ABC peptide transporter TAP that allows the transport of peptides from the cytosol into the ER (15). The key concept is that to this process, the MHC-I heterodimer is stabilized until a high-affinity peptide is incorporated into the peptide-binding groove. In most cases, cross-presentation is TAP- and proteasome-dependent (16), also called the *cytosolic* pathway. The proteasome is a self-compartmentalized, energy-dependent nanomachine that works as a protease to degrade misfolded, damaged, and inaccurately synthesized proteins (17). In the context of IFN-γ or DC maturation (18), the proteasome undergoes structural changes in its substrate-binding pockets that contribute to optimizing the quality and quantity of the generated peptides (19). Still, peptides generated by the proteasome may require further trimming by two ER-resident aminopeptidases (20). To make it more complex, proteasome-dependent, yet TAP-independent cross-presentation has been recently described, suggesting the existence of a still unidentified peptide transporter (Figure 1) (21).

A cross-presentation pathway referred to as *vacuolar* uses endolysosomal proteases to degrade internalized bacteria and other antigens, frequently particulated, in order to allow loading on MHC-I molecules recycled from the extracellular membrane (22). Also proteasome-derived peptides may enter the vacuolar pathway (23, 24). Data obtained from TAP^−/−^ DCs, that are unable to incorporate peptides via TAP into the ER, indicates that cross-presentation is still possible, though to a lesser extent (25).

Several questions remain unsolved, such as the mechanism by which antigens are exported from endosomes into the cytosol for proteasomal degradation (9), whether hybrid organelles resulting from the recruitment of TAP and the peptide-loading complex to phagosomes and endosomes exist (26–28), and if interconnected ER-phagosomes are involved in cross-presentation (29). Regardless of this issue, evidence indicates that the accumulation of antigen in endosomes with low (but steady) proteolytic and relatively high pH conditions favors cross-presentation (30–32). In this respect, it has been proposed that limited antigen degradation correlates with efficient cross-presentation (30). Primarily decreased proteolysis is found in the endocytic compartments of DCs compared to other phagocytes, which is due to low levels of lysosomal proteases, or decreased protease activity. These can be regulated by high pH present, or high activity levels of the NADPH oxidase 2 (NOX2) in endosomal and phagosomal compartments of DCs.

## Human DC Subsets and Antigen Cross-Presentation

Two main subsets of human DCs have been described: plasmacytoid DCs (pDCs) and myeloid DCs (mDCs, also known as conventional DCs). The majority of pDCs are located in the blood and their main function is the production of type I IFN upon microbial infection (33). Recent data also show that human pDCs are capable to cross-present antigens either derived from apoptotic cells (34) or when antigens are encapsulated in nanoparticles and targeted to specific uptake receptors expressed by pDCs (35). Next to pDCs, two major populations of mDCs can be identified in blood: BDCA1^+^/CD1c^+^ DCs and BDCA3^+^/CD141^+^ DCs. The BDCA3^+^ DCs are described as potent inducers of CD8^+^ T cell responses *in vitro* and *in vivo* (36–39); however, it is not yet clear how this capacity relates to the other human DC subsets (39–41). A recent publication showed that blood BDCA3^+^ DCs are more potent in cross-presentation compared to BDCA1^+^ DCs when antigens of necrotic cells or soluble antigen were given that ended up in late endosomes and lysosomes (41, 42). In contrast, when antigens were targeted to early endosomes, using antigens conjugated to an anti-CD40 monoclonal antibody, BDCA1^+^ DCs were as efficient at cross-presentation as BDCA3^+^ blood DCs. These results suggest that the capacity of DC subsets to cross-present is not intrinsic, but might also be determined by the route of antigen uptake and subsequent accumulation of the antigen in early endocytic compartments.

Due to the small number of mDCs in tissues, studies on human mDCs have been hampered, with the exception of the human skin. Based on the expression of CD1a and CD14, the human skin contains at least three main subsets of DCs: CD1a^+^/CD1c^+^ dermal DCs (dDCS), CD14^+^ dDCs, and CD1a^High^ epidermal Langerhans cells (LCs), which all migrate to the skin-draining lymph nodes upon activation (43). LCs and CD1a^+^ dDCs seem to be more efficient at cross-presentation, as compared to the CD14^+^ dDCs (44, 45). In addition to the three main populations of skin DCs, a minor BDCA3^High^CD14^−^CD111c^low-int^ subset of DCs is recently identified in human skin, lung, and liver. Parallel phenotypic analyses suggest that these cells are potentially related to blood BDCA3^+^ DCs. The skin BDCA3^High^ DCs have shown to be superior in cross-presentation of soluble antigens when compared to the other skin DC subsets, as well as compared to BDCA3^+^ DCs, BDCA1^+^/CD1c^+^ DCs, and CD14^+^ monocytes derived from blood (41). Care should be taken not to confuse the BDCA3^High^ skin DCs described by Haniffa et al. with the dermal BDCA3^+^CD14^+^ DCs described by Chu et al. (46). The latter are immunoregulatory tissue-resident DCs characterized by the constitutive secretion of IL-10 (46).

Altogether, findings on cross-presentation capacity of human DC subsets show that most subsets are capable to cross-present antigens. However, it becomes clear that other factors also influence the capacity to cross-present, like the antigen formulation, the mode of delivery, and the intracellular routing of the antigen, as well as the activation signals for the DCs.

## Factors Determining Cross-Presentation

The capacity of DC to cross-present antigens is not only dictated by characteristics of a given DC subset, but it starts to become clear that additional factors influence the cross-presentation capacity of these DC subsets as well. It must be mentioned however that most knowledge about human DC function is obtained from *in vitro* studies and thus may not fully reflect their behavior *in vivo*.

### Mode of antigen internalization

Antigens can be taken up by DCs via multiple mechanisms, including non-specific, receptor-independent processes, like pinocytosis and phagocytosis, or via specific, receptor-mediated processes such as uptake through CLRs, Fc receptors, and scavenger receptors. Blood BDCA3^+^ DCs are reported to be able to cross-present untargeted pp65 recombinant protein to a lesser extent than blood BDCA1/CD1c^+^ DCs *in vitro*. However, when the cells were stimulated with polyI:C, the BDCA3^+^ blood DCs became more potent to cross-present the pp65 protein compared to CD1c^+^ blood DCs (39). These results were confirmed by Mittag et al., who showed that CD1c^+^ blood DCs are more potent in cross-presenting soluble influenza protein without TLR stimulation, but in the presence of polyI:C the BDCA3^+^ blood DCs became more potent (47). Surprisingly, they also show that pDCs were able to cross-present soluble protein in the absence of polyI:C. Whether human blood-derived pDCs are capable to cross-present soluble proteins is questionable, since others provided evidence that pDCs were unable to cross-present soluble proteins in the presence and absence of TLR stimulation (48–50).

In addition, cross-presentation of NY-ESO-1 antigen administrated as antigen-antibody immune complexes (IC), allowing Fcγ receptor-mediated uptake, did not enhance antigen-specific CD8^+^ T cell responses by pDCs (50). In comparison, BDCA1^+^ blood DCs cross-presented the Fcγ receptor-targeting NY-ESO-1/IC more efficiently compared to the soluble protein formulation. Another study also showed that Fcγ receptor-mediated uptake of pp65-IC enhanced the cross-presentation capacity of both the BDCA1^+^ and BDCA3^+^ DCs compared to the uptake of HCMV pp65 protein (50, 51). These studies indicate that the mode of antigen internalization and antigen formulation have a profound impact on cross-presentation capacity.

Besides the uptake of antigen via Fcγ receptors, receptor-mediated uptake is also often studied using CLRs to stimulate antigen cross-presentation and CD8^+^ T cell responses. CLRs are a family of pattern-recognition receptors expressed by DCs and recognize various carbohydrate structures. Upon recognition and binding to the receptor, most CLRs respond by internalization and processing of the antigen (52). Their specific expression on certain DC subsets and the capacity to internalize antigens, make CLRs interesting targets to induce cross-presentation.

Targeting of antigen to the CLR DCIR, which is expressed by all human DC subsets tested, including LCs and blood mDCs and pDCs, resulted in improved cross-presentation by all subsets (53). Again, the blood mDC subset induced the highest percentages of tetramer-positive CD8^+^ T cells, indicative of a superior capacity to cross-present antigens, also when they are taken up in a receptor-mediated fashion. However, not all receptors show the same effects on antigen cross-presentation, as shown by Cohn et al. (42). Their study showed that BDCA3^+^ DCs were superior in cross-presentation of antigens taken up via the CLR DEC-205, which routes antigen to late endosomes and lysosomes, compared to BDCA1^+^ DCs and pDCs. However, when antigens were delivered to early endosomes through conjugation to CD40 or CD11c, BDCA1^+^ DCs and pDCs were as efficient in antigen cross-presentation as the BDCA3^+^ DCs (42). Furthermore, Chatterjee et al. have shown that targeting antigen to CD40 resulted in the most efficient cross-presentation in human moDCs and BDCA1^+^ DCs, despite the fact that CD40 was least efficient in antigen internalization compared to DEC-205 or mannose receptor (MR) (54). These results indicated that routing of antigen to more degradative, late endosomes, via DEC-205- or MR-mediated uptake, may have a negative effect on cross-presentation compared to antigen routing to early endosomes. Altogether, the results demonstrate that the intracellular routing of antigens is of importance for antigen cross-presentation. Thus, all human DC subsets seem to have the capacity to cross-present antigens, provided that the antigen is given in a suitable formulation under appropriate conditions.

### Antigen formulation

The antigen form and mode of delivery is crucial in determining the efficiency of cross-presentation. As DCs encounter antigens in many sizes and shapes, derived form various sources, multiple antigens might be differently handled by DCs, which might result in modification of the intracellular routing of antigen, thereby affecting the potency to cross-present. As described above, antigen can be soluble, as synthetic long peptides, protein, or it can be included in a pathogen/viral structure, as necrotic cells or as immune complex. Alternatively, antigens can be conjugated to antibodies specific for DC uptake receptors, or glycans that interact with CLRs. These different antigen formulations may affect the size of the antigen and receptor-targeting specificity, possibly affecting the type of DC that interacts with the antigen and the mode of uptake and intracellular routing.

To achieve and promote cross-presentation, different antigen formulations have been studied, such as nanoparticles, apoptotic cells or monoclonal antibodies, or glycans conjugated to antigens as discussed earlier. Targeting antigen to DNGR-1/CLEC9a, which expression in humans and mouse is restricted to CD8α^+^-like DCs (55), using PLGA nanoparticles conjugated to CLEC9a Moabs increased cross-presentation compared to isotype-control PLGA nanoparticles, implying that antigen uptake via CLEC9a enhances routing of the antigen to the cross-presentation machinery (53). Our own results show that targeting antigen to the CLR DC-SIGN using glycan- or antibody-modified liposomes results in enhanced cross-presentation capacity of DCs *in vitro* and *in vivo* (56). Furthermore, dendrimer technology has shown that a multivalent presentation of antigen, as well as particle size, enhances cross-presentation by DCs. Glycosylation of dendrimers enhances the DC-SIGN-mediated uptake of the particles, favoring enhanced CD4^+^ and CD8^+^ T cell responses (57).

There is evidence that also for LCs, the antigen formulation is crucial in order to allow cross-presentation by LCs. It has been shown that isolated human LCs were incapable to cross-present heat-inactivated measles virus, which is specifically taken up via Langerin (58). In contrast, others have shown that skin-derived human LCs were capable to cross-prime influenza-specific CD8^+^ T cells after targeting with an influenza protein conjugated to anti-Langerin antibodies (48), demonstrating that there is an inconsistency whether human LCs can cross-present or not and under which circumstances. Altogether, these findings demonstrate that the formulation of antigen (either small peptides or bigger particles, like viral- or bacterial-antigens, necrotic cells, and nanoparticles) has proven to have an influence on the cross-presentation capacity of various DC subsets.

### Adjuvants and DC maturation status

In general, DC maturation enhances the potency of DCs to cross-present antigen. A large set of TLR ligands are known that act as adjuvants and stimulate cross-presentation. Because each DC subset may express a specific set of TLR receptors, they may differently respond to TLR ligands, influencing the induction of cross-presentation. For example, isolated human LCs show increased cross-presentation of antigenic peptides in the presence of the TLR3 ligand polyI:C, whereas addition of the TLR4 adjuvant LPS or the TLR7/8 adjuvant R848 does not enhance the capacity to cross-present (Fehres et al., submitted). For instance the human skin, an attractive site for vaccination because it harbors many, easy-accessible DCs, is currently studied to identify suitable adjuvants to trigger and activate skin DCs for cross-priming. We and others have shown that intradermal administration of soluble TLR ligands does not induce DC maturation as observed with *in vitro* generated monocyte-derived DCs (59). The discrepancy between DC maturation after TLR activation *in vitro* and *in situ* might be caused by specific, local suppression within the skin microenvironment. Ideally, the adjuvant simultaneously stimulates several cell types, resulting in a mix of activated immune cells, cytokines, and chemokines at the vaccination site. Most promising into this respect seems Aldara, an FDA-approved immune response modifier skin cream, containing 5% of the TLR7 agonist imiquimod. Aldara is mostly used to treat non-melanoma skin tumors. Recently it was shown that application of Aldara cream results in inflammasome activation and IL-1 release by keratinocytes in naïve murine skin (60). This effect was mediated independent of TLR7 activation and attributed to isostearic acid, the major component of the vehicle. However, for induction of full inflammation, both imiquimod and the vehicle cream were shown to be required. Following topical application of Aldara skin cream to human skin explant, we observed enhanced migration and maturation of dermal DCs (Fehres et al., submitted). Combining the Aldara skin cream with Mart-1-peptide vaccination in human skin affected the migratory potential of CD14^+^ skin DC, which was associated with up-regulation of co-stimulatory molecules and increased activation and IFN-γ secretion of Mart-1-specific CD8^+^ T cells. Notably, the enhanced effects on DC and T cell activity were not observed when injecting soluble TLR7 and/or 8 ligands intradermally.

Besides being used as adjuvant in cancer vaccines, the aforementioned DC stimuli have also been used as stand-alone immunotherapeutics. It is anticipated that application of adjuvants at the tumor site reverses the immune-inhibitory phenotype of tumor-infiltrated DCs that ingest tumor antigens (TA), herewith restoring TA-specific T cell priming and anti-tumor immunity. An advantage of local delivery is a strong reduction in immune-related adverse events such as cytokine release syndrome and liver toxicity observed with systemic treatment. Indeed, topical application of the imiquimod containing cream led to residual tumors in 8% of patients in basal and squamous cell carcinoma patients (61). Furthermore, near tumor injection of low doses of agonistic anti-CD40 antibodies in a slow-release formulation was shown to activate TA-specific CD8^+^ T cells, which were able to act systemically and eradicate distant tumors (62). In addition, intra-tumoral injection of a TLR2/6 agonist spectacularly prolonged survival of pancreatic cancer patients with 9 months (63). The beneficial effects of TLR2/6 treatment were attributed to emergence of a strong immune response. Increased NK cytotoxic activity as well as elevated levels of TNF and IL-6 were noted.

Although soluble TLR ligands do not evoke strong maturation of skin DCs when injected into the skin as adjuvant, the discovery that tumor cells express TLRs has evoked interest in application of TLR agonists as monotherapy at the tumor site (64). Administration of a TLR3 agonist in melanoma lesions limits cell proliferation directly. Additionally, combined with a protein synthesis inhibitor even tumor cell death was induced (65).

The use of intradermal injected cytokines as immunostimulators has been explored (66, 67). In particular, granulocyte-macrophage colony-stimulating factor (GM-CSF) enhanced recruitment of DCs to the vaccine administration site, which ensures presentation of the administered TA by professional DCs and consequently priming of TA-specific T cells (66, 68). Furthermore, clinical trials have been conducted and/or are ongoing in which patients receive irradiated tumor cells genetically engineered to over-express GM-CSF (69). A small number of responses were demonstrated in Phase I trials in renal cell carcinoma and melanoma patients (68). However, in subsequent studies, GVAX monotherapy did not result in clinical responses. Indeed, the efficacy of GVAX might be improved by combining with immune check-point inhibitors, which aim to prevent inhibition of effector T cells and/or to silence Tregs. In murine pre-clinical models, GVAX combined with anti-CTLA-4 treatment enhanced efficacy and tumor regression in the B16 melanoma model, along with the presence of certain toxicities, such as skin depigmentation (70). Recently, a phase I study was completed showing dose escalation and safety, warranting further investigation of treating patients with this combination. Alternatively, GVAX has been combined with chemotherapeutic agents such as cyclophosphamide, which is currently being tested in clinical trials in metastatic melanoma patients. However, chemotherapy has been associated with immunosuppressive effects at standard doses, rendering issues related to dosing and timing of application critical.

The effect of GM-CSF may be further enhanced by co-administration of IL-2. Adjuvant activity has also been attributed to IL-2, which has been widely used in clinical trials and usually is administered systemically. However, in murine tumor models GM-CSF and IL-2 were shown to act synergistically when applied intradermal in emulsion along with a peptide, leading to improved and long-lasting peptide-specific CTL responses (66).

However, care should be taken using IL-2 as it may negatively impact on anti-cancer responses (e.g., promoting the accumulation and/or activation of Tregs). Recently, attention has focused on another cytokine belonging to the common gamma chain family: IL-21. IL-21 can exert potent anti-tumor effects due to its ability to induce and expand CD8^+^ CTLs and NK cells. Importantly, IL-21 suppresses FOXP3 expression and the expansion of regulatory T cells (Tregs). Recently, it has been shown that tumor-infiltrating lymphocytes expanded *ex vivo* with APCs engineered to secrete IL-21 performed better than those expanded in the presence of IL-2 (71). Moreover, the CD8^+^ T cells expanded in the presence of IL-21 exhibited a less differentiated, “young” phenotype. To date, there are no studies describing inclusion of IL-21 in therapeutic vaccines. Yet, promising results have been obtained *in vitro*: mature DCs transfected with IL-21 were superior in priming naïve CD8^+^ T cells than non-transfected DCs (72).

### Micromilieu rendering T cells dysfunctional

Both, chronic antigen expression and suboptimal priming in the tumor-environment renders TA-specific T cells dysfunctional. Chronic exposure to TA leads to exhausted T cells while suboptimal priming due to poor antigen presentation at tumor sites drives T cells into anergy (73, 74). These different aspects of T cell function can be discerned by addressing expression of specific sets of inhibitory receptors on TA-specific T cells. TA-specific CTLs present in peripheral blood lymphocytes (PBL) or at tumor sites have been shown to up-regulate PD-1 expression, which regulates their expansion (75–77). Next to PD-1, also the inhibitory receptors Tim-3 and LAG-3 can be upregulated on tumor-infiltrating T cells and serve as markers for exhausted T cells. By contrast, anergic T cells are characterized by BTLA expression (78). Notably, BTLA has been detected on spontaneous Mart-1- and NY-ESO1-specific CD8^+^ T cells in advanced melanoma patients (79, 80).

Expression levels of PD-1 on exhausted T cells correlate with inhibition of different aspects of CTL function (81). As blocking Abs display most affinity for PD-1^high^ expressing cells, functions inhibited due to low and/or intermediate PD-1 levels will not be regained (i.e., IL-2, TNF-α production and proliferation and cytotoxic activity, and IFN-γ production, respectively). The observation that PD-1 block does not alleviate the function of TA-specific CTLs on a per-cell basis argues in favor of combining this strategy with blocking other immune check-point inhibitors. Indeed, studies performed in patients and in mice with advanced melanoma showed that blockade of both PD-1 and Tim-3 acts synergistically to enhance TA-specific CD8^+^ T cell numbers and functions, resulting in decreased tumor growth (82–84). Likewise, combining Lag-3 blockade with PD-1 blockade may enhance activity of PD-1 blockade.

It has been shown that TA-specific CD8^+^ T cells exhibited variable levels of dysfunction, which correlated with a specific expression pattern of markers (80). BTLA blockade has been shown to act in concert with PD-1 and Tim-3 blockades to further enhance NY-ESO-1-specific CD8^+^ T cell expansion and function (80). The specific combination of inhibitory and anergy-related molecules might indicate a hierarchical loss of T cell function in patients with advanced melanoma in context of chronic antigen stimulation. Moreover, BTLA expression is inversely correlated with CD8 T cell maturation and thus anergic BTLA^+^ T cells are likely to represent young TA-specific CTLs. Recently, a positive association of CD8^+^ T cells expressing BTLA with clinical response to adoptive T cell therapy in late-stage melanoma patients has been suggested by Haymaker (85).

**Figure 2:**
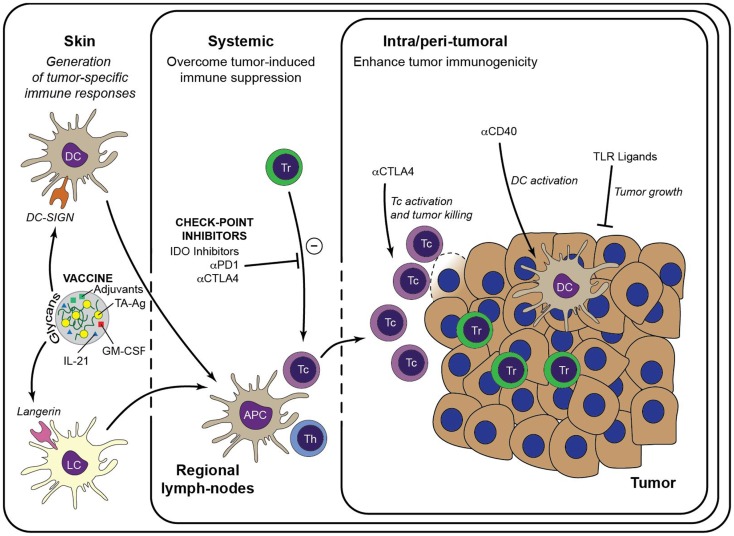
**Immunotherapeutic strategies to enhance anti-tumor immunity**. Generation of a large pool of effector TA-specific T cells is induced by the intradermal injection of anti-tumor vaccines. Targeting of the vaccine to a particular skin DC subset is facilitated by modification with specific glycans that bind either to DC-SIGN or Langerin. Subsequent vaccine internalization induces presentation of TA-Ag and maturation of the DCs. Matured DCs migrate to draining lymph nodes to prime TA-specific CD8^+^ T cells and CD4^+^ T helper cells, leading to a large pool of cytotoxic effector T cells that are capable to infiltrate the tumor lesion and lyse tumor cells. Priming of TA-specific T cells may be enhanced by inclusion of immunostimulators such as GM-CSF and IL-21 in the DC-targeting vaccine. Systemic or intra-tumoral administered check-point inhibitors, such as anti-PD-1 and anti-CTLA-4, release the break on the anti-tumor immune response by limiting the activity of suppressive Treg and alleviate the priming and/or function of TA-specific CTLs. Similarly, anti-tumor immunity may be enhanced by manipulation of the local micromilieu via administration of DC activating antibodies (e.g., anti-CD40) or of TLR ligands that act directly on the tumor cells. It is anticipated that these strategies may enhance the efficacy of DC-targeted vaccination. Tc, cytotoxic CD8^+^ T cell; Th, T helper cell.

Alternatively, these approaches may be even more active when combined with other agents that activate or inhibit key molecular regulators of T cell function, such as, for example, the tryptophan converting enzyme indoleamine-2,3-dioxygenase (IDO) and the membrane-bound CD39 and CD73 that breakdown arginase. IDO is highly expressed in both tumor cells and immune cells in the tumor-environment and implicated in inhibiting anti-tumor immunity by promoting the induction of anergic and/or regulatory T cells (86–89). Importantly, using pre-clinical animal melanoma models it was recently shown that IDO is responsible for mediating resistance to anti-CTLA-4 and anti-PD-1 therapy (90). Two IDO inhibitors have entered clinical trials: the tryptophan analog 1-methyl-tryptophan (1-MT) and the enzymatic inhibitor of IDO termed INCB024360. Both IDO inhibitors have been effective in pre-clinical models, attenuating tumor growth in wild type but not immuno-deficient mice (91, 92). INCB024360 has now entered Phase II trials, where it will be tested as a monotherapy in ovarian cancer and as a combination therapy with ipilimumab (anti-CTLA-4) for metastatic melanoma (Figure 2).

## Implications for Therapy Design – Future Directions

Our understanding in the mechanism of cross-presentation is crucial in the design of vaccination strategies aimed to induce protective immunity in the field of infectious diseases and cancer, which depends on the induction of both effector CD4^+^ and CD8^+^ T cells. Enhanced immune protection was obtained by long synthetic peptides compared to short peptides, which required cross-presentation of DC, resulting in long-lasting T cell stimulation that leads to the eradication of tumors (93, 94). Studies on improving cross-presentation-based vaccinations have emerged as a promising tool for immune intervention, based on many human *in vitro* studies and murine *in vivo* work. Strong focus on DC-targeting receptors *in vivo* that mediate endocytosis show potential of efficient induction of CD8^+^ T cell cross-priming, but can also lead to CD8^+^ T cell cross-tolerance. This fine tuning between the induction of immunity or tolerance is dictated by the various parameters that affect cross-presentation as mentioned under the Sections “Human DC Subsets and Antigen Cross-Presentation and Factors Determining Cross-Presentation,” the vaccine formulation, DC subset, receptor-targeting, endocytosis, and maturation stimuli. Many *in vivo* DC-targeting studies have been performed in mice that have demonstrated effective induction of tumor CD8^+^ effector T cell responses through targeting of CLRs, such as CD205, MR, CD207 (Langerin), CD209 (DC-SIGN), CLEC9A or other cell-surface receptors like integrins, HSP receptors, and glycolipids. In contrast, only a few of these DC-targeting vaccines have been tested in human clinical trails. Easy translation from mouse models to humans is complicated by the different expression levels of DC-targeting receptors and DC restriction and usage of TLRs between mouse and human. Moreover, still little is known on the cross-presenting capacity of human DC *in situ*. This has hampered the development of novel-targeted vaccination strategies for clinical applications, and is a complicated task to fulfill in the coming years. Highly effective DC-targeting therapies should overcome the mechanism of immune escape dictated by the tumor microenvironment. Therefore, combined regimens consisting of strategies to improve DC-induced T cell responses, increasing the frequency of anti-tumor T cells, reversing T cell exhaustion that stimulate trafficking to the tumor site, along with a blockade of immune-inhibitory pathways, all may be necessary to achieve clinical benefit for cancer patients.

## Conflict of Interest Statement

The authors declare that the research was conducted in the absence of any commercial or financial relationships that could be construed as a potential conflict of interest.
